# Enhancing Diamond Color Center Fluorescence via Optimized Configurations of Plasmonic Core–Shell Nanoresonator Dimers

**DOI:** 10.1021/acsomega.3c04902

**Published:** 2023-10-27

**Authors:** András Szenes, Dávid Imre Vass, Balázs Bánhelyi, Mária Csete

**Affiliations:** † Department of Optics and Quantum Electronics, University of Szeged, Dóm tér 9, Szeged 6720, Hungary; ‡ Wigner Research Centre for Physics, Konkoly-Thege Miklós út 29-33, Budapest 1121, Hungary; § Department of Computational Optimization, University of Szeged, Árpád tér 2, Szeged 6720, Hungary

## Abstract

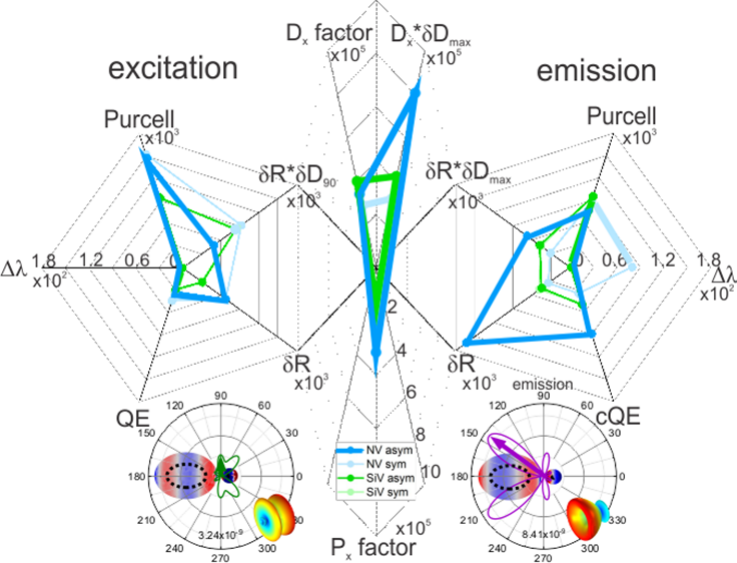

Numerical optimization of silica-metal core–shell nanoresonator dimer geometries was realized to maximize the fluorescence of the NV and SiV diamond color centers. The configurations combine the advantages stemming from the elongation and reduced metal volume of hollow spheroids and the wide tunability and good antenna efficiency due to hybridization of composite modes on the core–shell dimers. The optimized coupled dimers sustain plasmonic modes that maximize the fluorescence by ensuring the simultaneous enhancement of excitation and emission. Asymmetry is advantageous in terms of good enhancement with a compromised corrected quantum efficiency. The directional fluorescence can be significantly increased in the optimized asymmetrically coupled dimer configurations.

## Introduction

Recent approaches in quantum key distribution (QKD) and quantum information processing (QIP) rely on encoding qubits into the properties of single photons.^1^ Nonlinear elements offer scalability in next-generation quantum computers based on single-photon sources.^2^ However, efficient information encoding and transfer in the quantum states of light require high operation rates and high signal-to-noise ratios.

Diamond color centers, specifically nitrogen vacancy (NV) and silicon vacancy (SiV) color centers, are promising single-photon sources for QKD and QIP due to their reproducibility, scalability, and outstanding stability at room temperature. They exhibit narrow (1–5 nm) zero-phonon line emission in the near-infrared region, short lifetimes (1 and 25 ns), good intrinsic quantum efficiency (*QE*^0^), and remarkable spin properties, which facilitate spin-polarization entanglement even at room temperature.^3−6^

High extrinsic quantum efficiency (*QE*), indistinguishability, and polarization degree are crucial in quantum applications. These parameters can be improved using nanoresonators with a high quality factor (*Q*-factor) and low mode volume.^7,8^ Well-designed nanoresonators also improve the directivity of single-photon sources, while the large Purcell factor (Purcell)^9^ increases the achievable repetition rate via lifetime reduction.^1^ This is due to the localized surface plasmon resonance (LSPR) of metal nanoparticles that enhances the electric field and increases the local density of optical states (LDOS) in the proximity of the nanoparticles,^10−12^ allowing for tailored decay dynamics of fluorescent emitters including diamond color centers.^13,14^

Metal nanoshell structures support LSPR that is widely tunable via their shell thickness, which determines the hybridization of the primitive cavity and sphere plasmon resonances.^15,16^

Dimers composed of two individual metallic nanoparticles have further advantages in tailoring plasmon resonances, due to the strongly enhanced electric field in the interparticle nanogap, and quenching suppression via dark mode hybridization into bright antenna modes.^17−19^

As a consequence, core–shell dimers are promising to enhance fluorescence due to the combined inherent tunability, reduced metallic volume, boosted *E*-field, and suppressed quenching.^20−22^

Elongation of spherical metallic nanoparticles lifts the degeneracy of primitive plasmonic resonances corresponding to the orthogonal axes, resulting in transversal and longitudinal resonances with different frequencies depending on the nanoparticle axis ratio.^23^ Moreover, elongated particles with high curvature at their apex further enhance the *E*-field in the nanogap. Hence, layered, nonspherical nanoparticles arranged in dimers or patterns possess a colorful palette of coupled hybrid plasmonic modes, which can be tuned over a wide wavelength interval to achieve the desired fluorescence enhancement effect.^24,25^

Most of the previous studies have focused either on the excitation or emission enhancement alternatively, and only a few works were presented on simultaneously enhanced phenomena.^26−29^ In this study, we show numerically optimized ellipsoidal core–shell dimers, designed to simultaneously enhance the excitation and emission of SiV and NV color centers. Although the fabrication of the proposed silica-metal ellipsoidal core–shell dimer and color-center-implanted diamond slab assembly is experimentally challenging, there are recent experiments where silver nanoparticles embedded into diamond were created with high tunability and scalability by combining chemical vapor deposition, evaporation, and heating.^30^ In another structure, color centers were created by ion implantation with an accuracy of 5 nm.^31^ As a bottom-up approach, atomic force microscopy was used to assemble elongated core–shell nanoparticles^23,32^ into dimers with a high accuracy.^33^

## Methods

**Figure 1:**
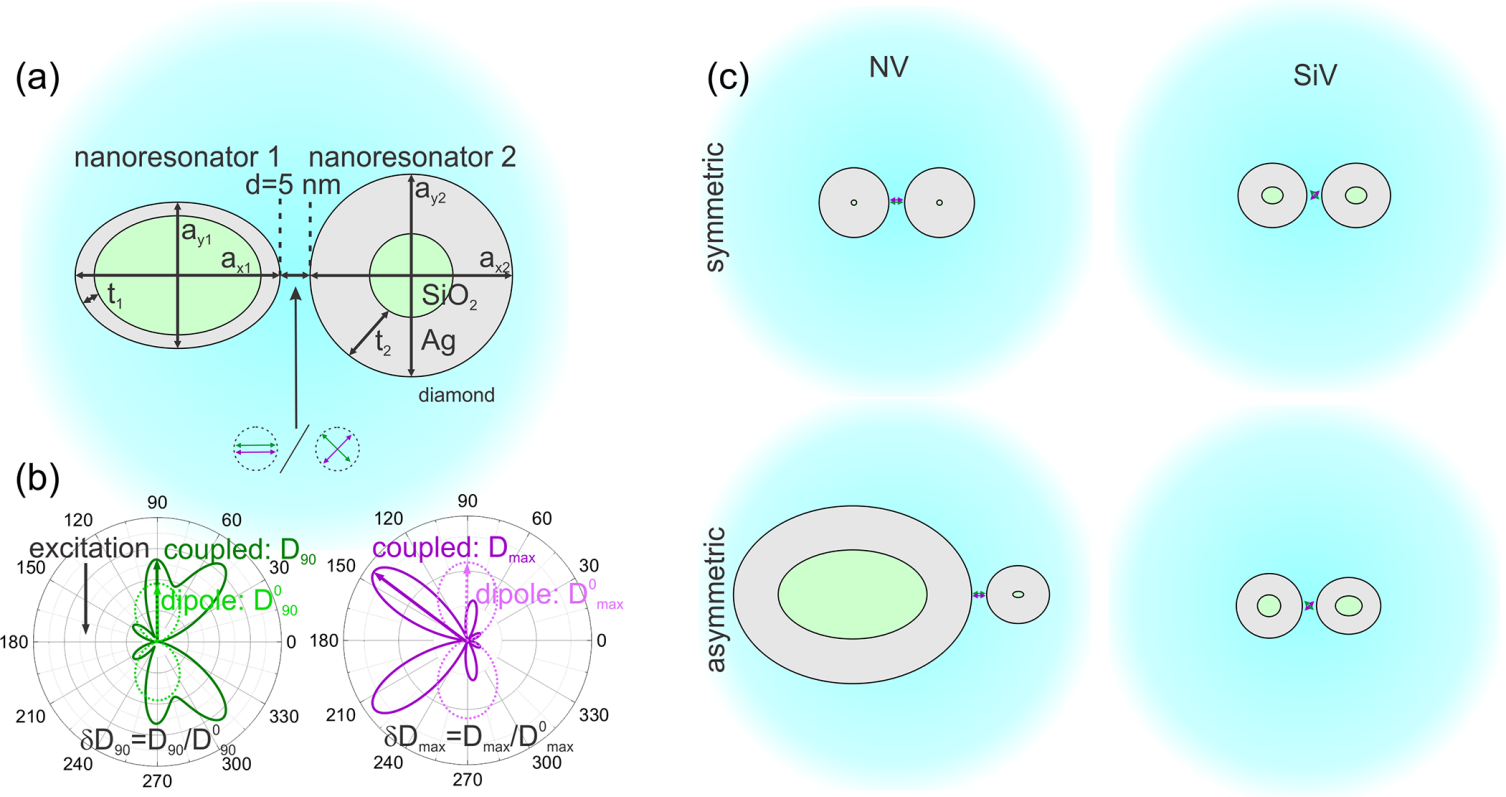
Schematics of silica-silver core–shell dimers. (a) Method of the geometry optimization and (b) explanation of directivity enhancement calculation at the excitation (left) and emission (right) wavelength: the lighter dashed lines show the power density radiation pattern of a dipole radiating in homogeneous diamond medium at the excitation (left) and emission (right). *D*^0^_90_ is the directivity of the dipole at a 90° polar angle, corresponding to the direction of excitation. *D*^0^_max_ is the maximum directivity of a single dipole at a given emission wavelength. The dark solid lines show the polar plots of the radiation pattern of a dipole coupled to a nanoresonator dimer. *D*_90_ is the directivity at 90° at the excitation, and *D*_max_ is the maximum directivity at the emission. The ratio of δ*D*_90_ = *D*_90_/*D*^0^_90_ corresponds to the enhancement of directionality at excitation, while δ*D*_max_ = *D*_max_/*D*^0^_max_ defines the enhancement of the maximum directivity at the emission. (c) Optimal configurations of the restricted symmetric/allowed asymmetric, NV and SiV color center coupled core–shell dimers. All indicated parameters are tuned separately.

To determine the optical response of the coupled diamond color center and nanoresonator dimer systems (Figure 1a), finite element method (FEM) simulations were performed using the RF module of COMSOL Multiphysics. By solving the full-wave Maxwell equations in the framework of classical electrodynamics, it is possible to characterize the excitation and emission enhancement separately, as well as their combined effect. To fully exploit the potential of FEM, we have defined quantities specifying excitation and emission enhancement. The evaluation of the fluorescence enhancing capabilities of the coupled core–shell nanoresonators are based on the method described in our previous works^27−29^ regarding core–shell dimer specific developments (see the Supporting Information for details). The new figure of merit functions are compatible with the nomenclature of our previous studies. Briefly, the *P*_*x*_ factor represents the coupled system’s total fluorescence enhancement and is the product of the radiative decay rate enhancements of excitation (δ*R*_exc_) and emission (δ*R*_em_): *P*_*x*_ factor = δ*R*_exc_·δ*R*_em_. In the projected fluorescence enhancement qualified by the *D*_*x*_ factor, the effect of the illumination direction is considered; thus, the *P*_*x*_ factor was weighed by the directivity enhancement along the direction of the excitation compared to free space in the presence of the dimer (δD_90_; Figure 1b): *D*_*x*_ factor = *P*_*x*_ factor·δD_90_.^34^ The directional fluorescence enhancement was also analyzed by evaluating the maximum directivity enhancement at the emission (δ*D*_max_; Figure 1b): *D*_*x*_ factor·δ*D*_max_. To quantify the losses of the diamond color center-nanoparticle assembly, the quantum efficiency of the coupled system was calculated as the ratio of radiative decay rate to the total decay rate (*QE* = γ_rad_/(γ_rad_ + γ_nonrad_)), and then it was corrected with the intrinsic quantum efficiency (*QE*^0^) of SiV color centers, to provide the apparent quantum efficiency (*cQE* = γ_rad_/(γ_rad_ + γ_nonrad_ + (1-*QE*^0^)/*QE*^0^)).

The nanoresonators forming the dimer were modeled as spheroidal silver or gold shells coating a small silica core (Figure 1a). By considering all criteria of the experimental feasibility, the plasmonic nanoresonators were assumed to be located in a thick diamond slab.

In the numerical model, the thick diamond slab was modeled as an infinite medium with bulk diamond properties; namely, a high (∼2.4) and wavelength-dependent refractive index medium surrounded the nanoresonator dimers. The wavelength-dependent material properties were obtained from tabulated data sets^35,36^ or using Cauchy formulas.^37^

The color center located on the dimer axis in the middle between the two constituting nanoparticles was modeled as an electric point dipole. The excitation peaks are at 532 nm, whereas the emission bands are centered around 650 and 737 nm in the case of the NV and SiV, respectively.^3−6,38,39^ The NV dipoles were oriented along the dimer axis, while the SiV dipoles were tilted by a 45° angle compared to the dimer axis, to balance the effect of a transient dipole moment orthogonality.^40,41^

The geometry optimization was performed with an in-house optimization algorithm.^42^ In this optimization, the *D*_*x*_ factor was the objective function. The *D*_*x*_ factor maximization inherently includes the simultaneous enhancement of both the excitation and emission phenomena; moreover, the in-coupling efficiency can also be controlled with the directivity.

During the optimization, the outer shell and inner core axes of the ellipsoids of constituting nanoresonators were tuned (Figure 1a). The interparticle distance was fixed at 10 nm to ensure a large *E*-field enhancement but to avoid quantum effects. Two different configurations were inspected: the constituting nanoresonators’ geometry was forced to be identical (symmetric), or the nanoresonators’ geometry was varied independently (asymmetric). The lower bound of the axis length was 12 nm, while the maximum allowed value was 160 nm for both orthogonal directions. The minimum allowed size of cores corresponded to 2 nm diameter.

From the calculated spectra, the plasmonic resonance detuning from the target wavelengths, the quantum efficiency, Purcell factor, excitation, and emission enhancements as well as the *P*_*x*_ and *D*_*x*_ factors and the directional fluorescence enhancement were determined (provided in Supporting Information Tables S1–S4). The far-field radiation patterns as a function of the φ polar angle were plotted, and the directivity enhancements were extracted.

**Figure 2:**
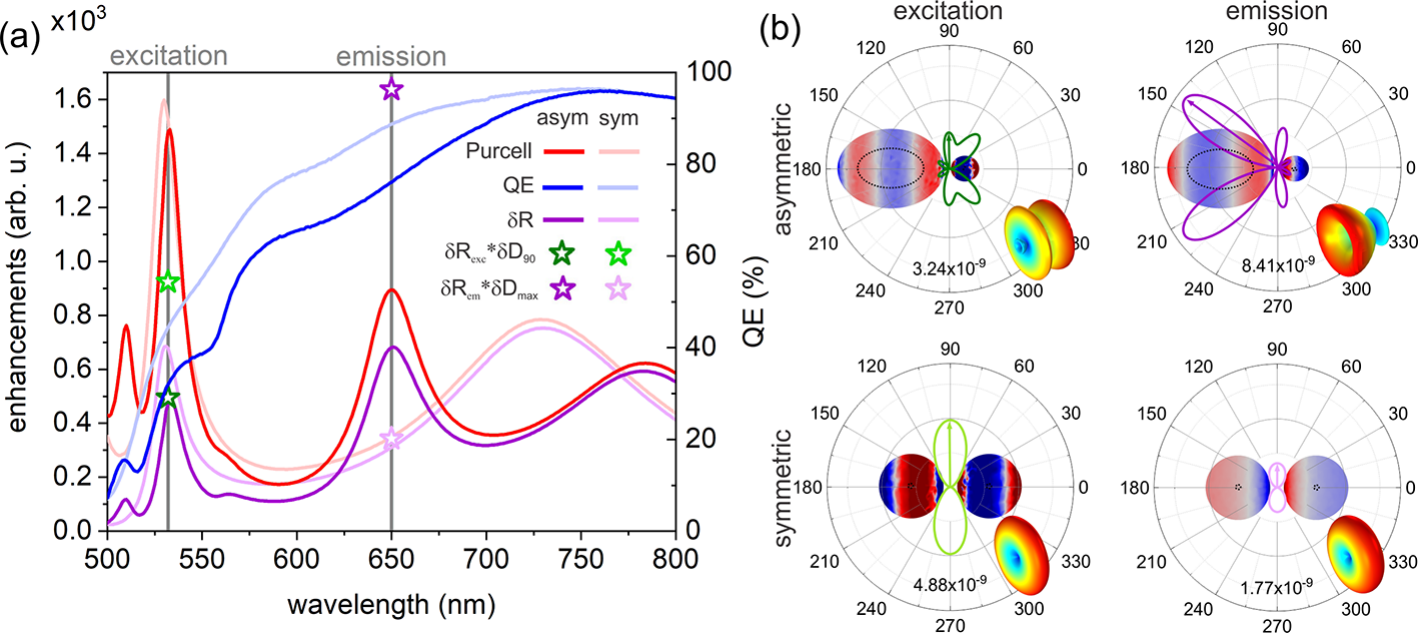
Optical response of optimized Ag–NV core–shell dimer configurations. (a) Enhancement and coupled antenna efficiency spectra. Purcell, Purcell factor; *QE*, quantum efficiency; and δR, radiative rate enhancement spectrum of the coupled system. The star-shaped symbols show the radiative rate enhancement multiplied by the directivity enhancement at the excitation and emission and define the excitation enhancement (green) and directional emission enhancement (violet), respectively. The darker colors correspond to the asymmetric configuration. (b) Charge distribution on the nanoantennae (blue is the negative minimum, while red is the positive maximum charge density) and polar angle (*φ*) distribution of the power density radiated into the far-field by coupled dipoles at the excitation (left) and emission (right). The lobe direction used for directivity calculation is marked with an arrow, and the maximal power density radiated into this direction is indicated at the bottom of the plot. For better visualization, the 3D radiation pattern with rotational symmetry is also shown in the insets.

Through the optimization of silver dimers, it was considered whether the allowed asymmetric geometry is advantageous in terms of the total, projected, and directional fluorescence enhancement (*P*_*x*_ factor, *D*_*x*_ factor, and *D*_x_·δ*D*_max_) (Tables S1 and S2 in the Supporting Information). The charge and radiation field distributions, as well as the spectral responses of the optimal coupled dimers, were analyzed (Figure 2). To validate and benchmark the FEM method, reproduction of analytical and experimental data is also provided in the Supporting Information (see Section 3 and Figures S6 and S7 of the Supporting Information).

## Results and Discussion

When the NV color center is coupled to an asymmetric silver nanoresonator dimer, the Purcell factor spectrum indicates four resonances in the inspected wavelength interval. The global maximum has a value of 1.5 × 10^3^ and coincides with the excitation wavelength (Figure 2a). A considerably smaller (0.9 × 10^3^) and wider resonance peak (i.e., smaller *Q*-factor) is tuned to the emission wavelength (650 nm) of the NV color center. The *QE* spectrum indicates a broad band of good antenna efficiency, peaking above 750 nm and resulting in a 76% *cQE* at the 650 nm emission wavelength, which corresponds to a 14% decrease compared to the 90% intrinsic *QE*^0^ of NV. Modulations in the *QE* spectrum indicate superimposed bright plasmonic modes neighboring the numerous resonances in the Purcell factor spectrum. The δ*R* spectra follow the Purcell factor spectra (Figure. 2a). According to the large projected excitation enhancement, related lifetime reduction, and accompanying large antenna efficiency, both the excitation (δ*R*_exc_·δ*D*_90_ = 5.0 × 10^2^) and emission (δ*R*_em_ = 6.8 × 10^2^) phenomena are resonantly enhanced.

The optimal dimer geometry is strongly asymmetric with elongated hollow spheroids of different volumes (Figure 1b). This asymmetry enables the enhancement of multipolar composite modes, leading to multiple resonance peaks in the Purcell factor spectrum. Both the excitation and emission are accompanied by multipolar composite modes. The excitation shows 2 × λ/2 – 1 × λ/2 modes with a parallel surface dipole around the nanogap. In comparison, a lower-order resonance enhances the emission without localized charge around the nanogap. Despite the multipolar nonradiative nature of modes, high quantum efficiency is obtained due to the plasmonic modes’ hybridization and allowing an increased radiative component.^17^ The coupled system’s radiation patterns differ from a simple radiating dipole in accordance with the multipolar resonant modes and the strongly asymmetric geometry. The radiation field distribution is associated with the coupled multipoles and uncovers the relative strength and polarity of the plasmonic modes and the impact of the coupling between the color center and antenna, as well as the directivity of emitted fluorescence radiation. At the excitation, the radiation pattern is multipolar, indicating higher-order plasmonic modes. At the emission, a less uniform distribution of higher-order modes is directed toward the larger spheroid nanoresonator. This radiation is more directed with a maximum directivity of *D*_max_ = 3.62 toward *φ* = 144°. The *P*_*x*_ factor = 3.2 × 10^5^ and *D*_*x*_ factor = 3.4 × 10^5^, along with the 2.4-fold maximum directivity improvement at the emission, indicate a significantly (8.1 × 10^5^) improved directional fluorescence.

Restricted symmetric configuration of silver core–shell dimers leads to a smaller fluorescence enhancement. The degree of Purcell factor tunability is reduced; namely, only two maxima appear, resulting in resonant excitation and off-resonant emission enhancement. The *QE* spectra’s FWHM and *cQE* achieved at 650 nm are larger (89%) than those in the asymmetric dimer. The induced modes allow for higher excitation enhancement but more significantly lower emission enhancement. The optimal geometry consists of almost solid spherical nanoresonators, which explains the low number and low tunability of the excited plasmonic modes. Symmetrical antenna modes of 1 × λ/2 – 1 × λ/2 are excited with/without an accompanying surface dipole at the excitation/emission wavelength. The symmetric configuration exhibits a dipole-like far-field radiation pattern indicating radiative composite modes both at excitation and emission, with slightly enhanced maximum directivity (*D*_max_ = 1.67). These result in smaller total and projected fluorescence enhancement (*P*_*x*_ factor = 2.1 × 10^5^ and *D*_*x*_ factor = 2.9 × 10^5^) along with a 1.1-fold directivity improvement and less improved directional fluorescence (3.2 × 10^5^) compared to the asymmetric counterpart.

Silica-gold core–shell dimer configurations optimized for NV color center fluorescence exhibit orders of magnitude lower *P*_*x*_ factor, *D_x_* factor, and directional fluorescence enhancement because of the stronger material limits, resulting in limited excitation improvement, weaker emission enhancement, lower quantum efficiency, and less improved directionality in symmetric and asymmetric dimers, compared to the optimized silica-silver core–shell counterparts (Figure S1 in the Supporting Information).

**Figure 3:**
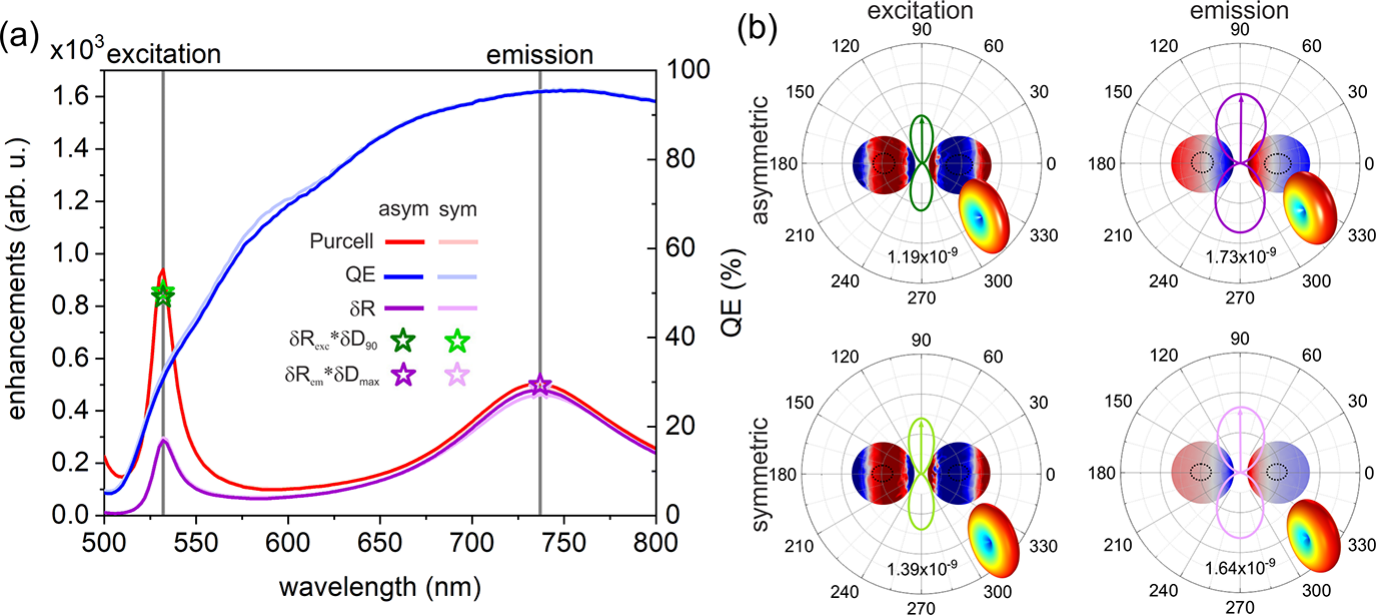
Optical response of optimized Ag–SiV core–shell dimer configurations. (a) Enhancement and coupled antenna efficiency spectra. Purcell, Purcell factor; *QE*, quantum efficiency; and δ*R*, radiative rate enhancement spectrum of the coupled system. The star-shaped symbols show the radiative rate enhancement multiplied by the directivity enhancement at the excitation and emission and define the excitation enhancement (green) and directional emission enhancement (violet), respectively. The darker colors correspond to the asymmetric configuration. (b) Charge distribution on the nanoantennae (blue is negative minimum, while red is positive maximum charge density) and polar angle (φ) distribution of the power density radiated into the far-field by coupled dipoles at the excitation (left) and emission (right); the lobe direction used for directivity calculation is marked with an arrow, and the maximal power density radiated into this direction is indicated at the bottom of the plot. For better visualization, the 3D radiation pattern with rotational symmetry is also shown in the insets.

The SiV color center coupled with asymmetric silica-silver core–shell nanoresonator dimers has also been optimized to maximize the projected fluorescence enhancement. The Purcell factor spectrum exhibits two resonance maxima exactly at the excitation (532 nm) and emission (737 nm) wavelengths, indicating a doubly resonant geometry (Figure 3). The resonance at the excitation is considerably more intense (0.9 × 10^3^), its FWHM is also smaller, indicating a high *Q*-factor, while the emission is enhanced via a less intense (0.5 × 10^3^) and broader resonance, indicating a lower *Q*-factor, i.e., faster energy decay. Both resonances are radiative in nature based on the *QE* spectrum.

At the emission wavelength, the nanoresonator allows for a 500-fold reduction in lifetime and a 95% cQE corresponding to a 9.5-fold increase compared to the intrinsic quantum efficiency of the SiV color center. The projected excitation enhancement exceeds the radiative rate and the directional emission enhancement at the emission, despite the three times larger *QE* at 737 nm. Namely, the optimized configuration results in δ*R*_exc_·δ*D*_90_ = 834-fold projected excitation enhancement, δ*R*_em_ = 479-fold radiative rate enhancement at the emission, and 499-fold directional fluorescence enhancement (*D*_*x*_·δ*D*_max_).

Both components are almost spherical in the optimal asymmetric silica-silver core–shell dimer geometry. The intermediate shell thicknesses and almost identical composing nanoresonators suggest a preference for symmetry with slight symmetry breaking despite the tighter restrictions. The almost spherical nanoresonators and symmetric components explain the few resonance maxima in the enhancement spectra. The excitation is enhanced via a 1 × λ/2 – 1 × λ/2 antenna mode accompanied by a surface charge separation around the nanogap. Its dipole-like far-field radiation pattern proves that the higher-order individual plasmonic modes hybridize to a radiating antenna mode. The emission is efficiently enhanced by the lowest-order dipolar–dipolar antenna mode with a large radiative component, as the *QE* spectrum indicates. Compared to the excitation, the resonance at the emission is less intense, which is in accordance with the smaller Purcell factor and a smaller amount of accumulated charge. The larger radiative rate enhancement correlates with the stronger dipole-like radiation pattern. The maximum directivity *(D*_max_ = 1.57) is slightly larger than a dipole in homogeneous medium (1.50); moreover, the direction of the radiation deviates from *φ* = 90° by only 1° due to the nearly symmetrical geometry. These result in smaller total and larger projected fluorescence enhancement (*P*_*x*_ factor = 1.38 × 10^5^ and *D*_*x*_ factor = 4.0 × 10^5^) compared to the NV center-coupled asymmetric dimer. Finally, the smallest 1.04-fold maximum directivity enhancement results in an intermediately improved directional fluorescence (*D*_*x*_·δ*D*_max_ = 4.2 × 10^5^) compared to the NV center-coupled symmetric and asymmetric dimers.

By requiring symmetry, the dimensions of the symmetric nanoresonators are very similar to those composing the optimal asymmetric dimer, resulting in almost identical enhancement spectra, quantum efficiency values, and far-field radiation patterns. As a result of lower tunability, the symmetric dimer has a smaller Purcell factor; however, the quantum efficiencies are slightly improved both at the excitation and emission. The combined effect of these quantities is that the (projected) excitation rate enhancement is larger but the (directional) emission rate enhancement is smaller. The latter is more dominant, thereby resulting in a 1% smaller total and projected fluorescence enhancement in the symmetric system compared to that in its asymmetric counterpart. These *P*_*x*_ factor = 1.37 × 10^5^ and *D*_*x*_ factor = 3.9 × 10^5^, along with a slightly larger 1.05-fold maximum directivity enhancement that results in *D*_max_ = 1.58 maximal directivity, indicate intermediately improved directional fluorescence (*D*_*x*_·δ*D*_max_ = 4.1 × 10^5^), which is also slightly smaller than in the asymmetric counterpart.

Orders of magnitude lower *P*_*x*_ factor and *D*_*x*_ factor can be achieved with silica-gold core–shell dimer nanoresonators along with slightly better/less improved directivities in asymmetric/symmetric dimers (Figure S2 in the Supporting Information). The larger material losses of gold, especially at 532 nm, have highly limiting effects in the case of SiV enhancement with gold as well.

SiV-core–shell systems offer better spectral tuning due to the larger emission wavelength; however, the lack of emitter orientation constraint is advantageous for NV color centers.

**Figure 4:**
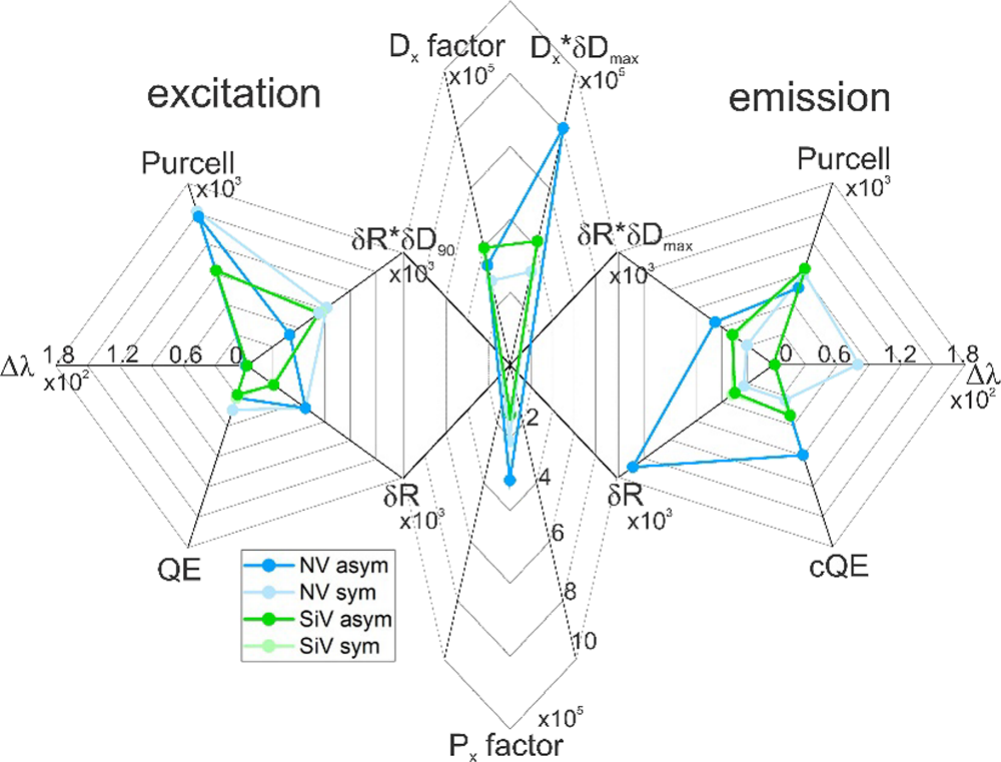
Comparison of optimized silica-silver core–shell dimers. The plot is separated into three parts: excitation (left), emission (right), and total fluorescence (middle). The excitation and emission parts describe the different readout quantities for the optimized configurations under study for a color center radiating at the excitation and emission wavelengths, thus qualifying the enhancement of the two processes. Purcell, Purcell factor; Δλ, detuning of plasmonic resonance spectrally near the central wavelength of excitation and emission in nanometers; *QE* (*cQE*), (corrected) quantum efficiency of the coupled system; δ*R*, radiative enhancement of radiating dipole; δ*R*·δ*D*_90/max_, radiative rate enhancement multiplied by the maximum directivity enhancement. *P*_*x*_ factor = δ*R*_exc_·δ*R*_em_ is the total fluorescence enhancement, *D*_*x*_ factor = *P*_*x*_ factor·δ*D*_90_ is the projected fluorescence enhancement, and *D*_*x*_·δ*D*_max_ = *D*_*x*_ factor·δ*D*_max_ is the directional fluorescence enhancement.

The most significant difference between the two asymmetric dimers optimized to maximize NV and SiV fluorescence is that the coupled NV-silica-silver dimer configuration is highly asymmetric, while the SiV-silica-silver dimer configuration is nearly symmetric. As a result, the NV-silica-silver dimer exhibits a multipolar charge distribution and an asymmetric far-field radiation pattern. The strongly asymmetric geometry also leads to more Purcell resonances, due to lifted degeneracy and selection rules, for the NV-asymmetric-silica-silver dimer. The radiative plasmonic modes are well tuned to excitation and emission in both NV- and SiV-coupled asymmetric configurations. A larger Purcell factor is ensured by the parallelism of the dipole and dimer axis orientation in coupled NV-silica-silver dimers (Figure 4, except at the emission in the symmetric case). The broad band of the quantum efficiency is peaked around 737 nm in both NV- and SiV-coupled silica-silver dimers with a similar FWHM. Consequently, a similar 31–32% *QE* and by a factor of 1.25× larger cQE are achievable with the coupled SiV-asymmetric-silica-silver dimer at the excitation and emission, respectively. With the coupled SiV- asymmetric-silica-silver system, a 1.2× larger projected fluorescence enhancement (*D*_*x*_ factor) is reachable, originating from the significantly larger projected excitation enhancement. Although the SiV-silica-silver system’s more symmetric geometry is experimentally more realizable, its maximal emission directivity is significantly smaller than that of the coupled NV-asymmetric-silica-silver dimer. The larger emission directivity overcompensates for the small directivity at the excitation, thereby resulting in the highest directional fluorescence enhancement in the coupled NV-asymmetric-silica-silver dimer.

When the *P*_*x*_ factor was selected as an objective function instead of the *D*_*x*_ factor, the relation between gold and silver held similarly. However, the relation between coupled SiV- and NV-asymmetric-silica-silver core–shell dimers is reversed in terms of the total fluorescence enhancement (Figures S3 and S4 in the Supporting Information).

Comparing the two optimized symmetric configurations, the NV-silica-silver dimer has larger quantities related to excitation (except the directivity enhancement), while the SiV-silver-silica dimer is better at the emission (except the directivity enhancement). Symmetric dimers maximize the color center fluorescence by improving their excitation. The NV center’s directed excitation is enhanced almost three times as much, while the SiV excitation is enhanced almost two times more than its emission. The relations between fluorescence enhancements and quantum efficiencies are different from those in asymmetric systems: smaller *QE*, δ*D*_max_, and *P*_*x*_ factor are obtained for SiV, while the *cQE*, *D*_*x*_ factor, and *D*_*x*_·δ*D*_max_ are higher. Moreover, the directional fluorescence enhancement is also larger for the SiV color center in the symmetric case due to comparable maximum directivity enhancements for both color centers.

## Conclusions

Silica-metal core–shell nanoresonator dimer geometries were numerically optimized successfully to maximize the fluorescence of the NV and SiV diamond color centers. The advantages of the smaller metal volume in hollow spheroids, hybridization of composite modes on the nanoshell, and wider tunability via dimers were combined in this study. A detailed analysis has shown that spheroidal core–shell geometries sustaining a few plasmonic modes are capable of maximizing the *D*_*x*_ factor (qualifying the projected fluorescence enhancement) by ensuring that phenomena at the target wavelengths are resonantly enhanced (except for the emission of the NV-symmetric-silica-silver dimer, which is off-resonantly enhanced). The allowed asymmetry was always advantageous for fluorescence enhancement maximization, but it was accompanied by a smaller corrected quantum efficiency compared to the symmetric counterpart dimers. The emission was always enhanced by the lowest-order bonding dipole–dipole mode. In the case of the strongly asymmetric geometry, the dipolar surface charge distribution is accompanied by a charge accumulation around the nanogap at the excitation, which is induced by the smaller nanoresonator dimer component. In the case of nanoresonators optimized to reach the maximal SiV enhancement, the allowed asymmetry results in a slightly adjusted geometry compared with the configuration determined via restricted symmetric optimization. The NV color center’s projected fluorescence enhancement and directional fluorescence enhancement can be increased by a factor of 3.4 × 10^5^ and 8.1 × 10^5^ in the optimized asymmetric configuration, respectively, and are accompanied by a corrected quantum efficiency of 76%. The SiV can be better enhanced than NV, considering the *D*_*x*_ factor: its projected fluorescence can be increased by a factor of 4.0 × 10^5^ and its corrected quantum efficiency (95%) is improved by a factor of 9.5 in the optimized asymmetric configuration. In contrast, the directional fluorescence enhancement is smaller in the case of SiV (*D*_*x*_·δ*D*_max_ = 4.2 × 10^5^).

## Supporting Information Available

Detailed numerical analysis of the optimized silica-gold core–shell dimers, inspection of the impact of the cQE criterion on the achievable total fluorescence enhancement and system characteristic, resistive loss spectra of the constituting nanoparticles of optimized dimers, and validation of the numerical method (PDF)
