# Supplementary material for: Enhancing Diamond Color Center Fluorescence via Optimized Configurations of Plasmonic Core–Shell Nanoresonator Dimers
Source: ACS Omega. 2023 Oct 27;8(44):41356–62. doi: 10.1021/acsomega.3c04902 (PMC10633910; doi:10.1021/acsomega.3c04902)
Supplement: Supplementary file 1 — ao3c04902_si_001.pdf [file ao3c04902_si_001.pdf]

Enhancing diamond color center fluorescence via optimized configurations of plasmonic core - shell nanoresonator dimers

András Szenes[1,2], Dávid Vass[1,2], Balázs Bánhelyi[2,3], Mária Csete[1,2,]*

[1]*Department of Optics and Quantum Electronics, University of Szeged, Dóm tér 9, Szeged 6720, Hungary*

[2]*Wigner Research Centre for Physics, Konkoly-Thege Miklós út 29-33., Budapest, 1121, Hungary*

[3]*Department of Computational Optimization, University of Szeged, Árpád tér 2, Szeged 6720, Hungary*

*mcsete@physx.u-szeged.hu

1. Methods

The impact of the plasmonic resonance is characterized by the Purcell factor [S1] of the color center, which is the enhancement of the total emitter decay rate: $Purcell = ((\gamma_{rad} + \gamma_{non\text{-}rad})/(\gamma^0_{rad} + \gamma^0_{non\text{-}rad}))$. The total decay rate enhancement can be calculated as the ratio of total power dissipation of the emitter in inhomogeneous and homogeneous medium: $\delta\gamma = ((P_{rad} + P_{non\text{-}rad}) /((P^0_{rad} + P^0_{non\text{-}rad}))$. The antenna efficiency of the coupled system is calculated as the ratio of the radiative and total decay rates: $QE = (\gamma_{rad}/(\gamma_{rad} + \gamma_{non\text{-}rad}) = P_{rad}/(P_{rad} + P_{non\text{-}rad}))$. The radiative rate enhancement can be computed as $\delta R_{em} = Purcell \times QE$.
At the emission the antenna efficiency was corrected with the QE^0 = 90% and 10% intrinsic quantum efficiency of the NV and SiV color centers, respectively [S2, S3], as $cQE = \gamma_{rad}/(\gamma_{rad}+\gamma_{non\text{-}rad}+\gamma^0_{rad}\cdot(1\text{-}QE^0)/QE^0)$ [S4]. The excitation enhancement ($\delta\gamma_{exc}$) equals to the near-field enhancement of the incoming electromagnetic field projected onto the dipole oscillation direction at the emitter position: $\delta\gamma_{exc} = |\boldsymbol{E}\cdot\boldsymbol{p}|/|\boldsymbol{E}^0\cdot\boldsymbol{p}|$, where $\boldsymbol{p}$ is the transition dipole moment of the emitter, $\boldsymbol{E}^0$ and $\boldsymbol{E}$ are the excitation field at the dipole position in a homogeneous diamond medium and in the presence of nanoresonators, respectively.

For efficient optimization it is advantageous to use the analogy provided by the reciprocity theorem [S5] between $|\boldsymbol{E}\cdot\boldsymbol{p}|/|\boldsymbol{E}^0\cdot\boldsymbol{p}|$ and δR_{exc}. The latter equals to the radiative decay rate enhancement (emitted power enhancement) of an imaginary dipole radiating at the wavelength of excitation (P_{exc}/P^0_{exc}). This excitation rate enhancement should be weighted by the directivity modification ($\delta D_{90} = D_{90}/D^0_{90}$) of the coupled system in the direction of illumination in a homogeneous environment to consider the specific direction of the dipole to be excited such as $\delta\gamma_{exc} = \delta R_{exc}\times\delta D_{90}$.

The emission enhancement is calculated as the radiative decay rate enhancement of a dipole radiating at the wavelength of emission: $\delta R_{em} = \gamma_{em}/\gamma^0_{em} = P_{em}/P^0_{em}$, where P_{em} and P^0_{em} is the total radiated power of the system in the presence of the nanoresonator and in a homogeneous medium, respectively. The directivity of the emission (D_{max}) was also considered, by calculating the maximal directivity enhancement of the coupled system compared to the dipole in the homogeneous diamond medium ($\delta D_{max} = D_{max}/D^0_{max}$). This directionality modification (δD_{max}) was then multiplied by the projected fluorescence enhancement ($D_x = \delta R_{exc}\times\delta D_{90}\times\delta R_{em}$) and the product was nominated as directional fluorescence enhancement ($D_x\times\delta D_{max}$)

2. Silica-gold core-shell dimers

The optimization for NV and SiV projected fluorescence maximization was performed for silica-gold core-shell dimers as well, and the corresponding spectra, surface charge-distribution and far-field radiation distributions, antenna efficiency as well as the total decay rate (*Purcell* factor), radiative rate and emission directivity enhancements (Tables S1-S4) were determined. The optimal symmetric and asymmetric coupled NV - silica-gold core-shell dimers have high *Purcell* factors both at the excitation and emission wavelengths, although the position of the maxima indicates that both processes are slightly off-resonantly enhanced (Figure S1). The quantum efficiency spectra show that below 600 nm, the excited modes are strongly non-radiative, while at the emission the *cQE* values are around 20%. As a result, only the emission enhancement contributes to the total, projected and directional fluorescence enhancement in case of gold dimers.

At 532 nm both for symmetric and asymmetric dimers only a local charge separation is noticeable at the nanogap which, together with the low quantum efficiency and broad Purcell factor maximum, indicates the excitation of a plasmonic pseudomode. In contrast, the emission can be efficiently enhanced by the lowest-order dipole-dipole modes, which are accompanied by a local surface charge separation in the vicinity of the gap of the asymmetric dimer. The far-field radiation pattern of the asymmetric system at the emission deviates from the typical dipolar character, which is the result of the coupled dipole modes, induced on the components of the strongly asymmetric dimer. The achieved D_x factors are 100 and 77 for asymmetric and symmetric systems with 21% and 24% *cQE*, respectively. These fluorescence rate enhancements are orders of magnitude smaller than the values achievable with silica-silver composing core-shell nanoresonators. Although, significant (slight) maximum directivity enhancement was achieved in case of the asymmetric (symmetric) geometry, the directional fluorescence enhancement is in the same orders of magnitude as the total and projected enhancement: $D_x{\cdot}\delta D_{max}$ = 167 (80).

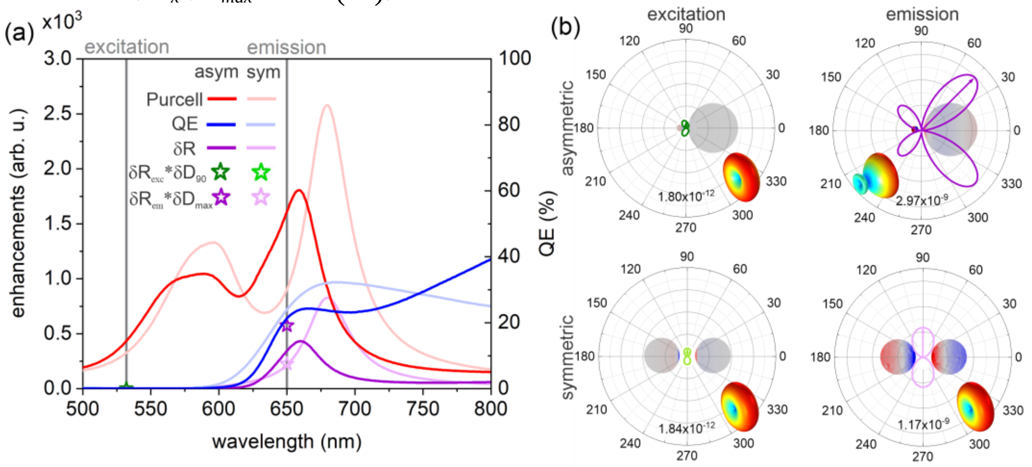

Figure S1. Optical response of optimized NV coupled silica-Au core-shell dimer configurations. (a) Enhancement and coupled antenna efficiency spectra. (b) Charge distribution on nanoantennae and polar angle (φ) distribution of power density radiated into far-field, and the 3D radiation pattern with rotational symmetry (the lobe direction used for directivity calculation is marked with arrow and the maximal power density radiated into this direction is indicated at the bottom of the plot).

The optical responses in optimized coupled SiV-silica-gold core-shell dimers are similar in many aspects to coupled NV-silica-gold core-shell dimers: high Purcell factors are achieved at excitation and emission wavelengths, but these are accompanied by very low quantum efficiency below 600 nm (Figure S2). Therefore, only the emission enhancement contributes to the total, projected and directional fluorescence enhancement.

By examining the charge distributions, it can be seen that only a pseudomode can be excited at the excitation wavelength due to interband transitions, while the emission can be enhanced by the lowest order antenna modes, both in symmetric and asymmetric cases. In the highly asymmetric case, the dipole-dipole modes are accompanied by a surface charge separation localized around the nanogap. At the excitation, the far-field radiation distribution is governed by the emitter orientation, which suggests a very weak coupling between the color center and the nanoresonator dimer. Deviation from the dipole characteristic is again observed at the emission in the asymmetric case, due to the coupled dipole modes induced on the composing nanoresonators. The achieved D_x factor is 301 by allowing asymmetry at the cost of reducing *cQE* to 37%, while it is smaller 175 (along with larger 60% *cQE*) in the case of a symmetric dimer. The achieved D_x factors are orders of magnitude smaller compared to the silica-silver core-shell counterpart dimers, alongside the significantly lower quantum efficiency and directivity, except the δD_{max} at the emission in the SiV coupled asymmetric dimers. The maximum directivity enhancement promotes to reach larger directional fluorescence enhancement of 509 and 181 in case of asymmetric and symmetric configurations, respectively, however it is still orders of magnitude smaller than the enhancements with SiV - silica-silver core-shell dimers. Based on the comparison of NV and SiV color centers, it can be concluded that SiV can be more efficiently enhanced with silica-gold core-shell dimers than the NV color center, but with similar directivities. It correlates with the higher quantum efficiency, which is the result of the lower metal losses at the larger emission wavelength.

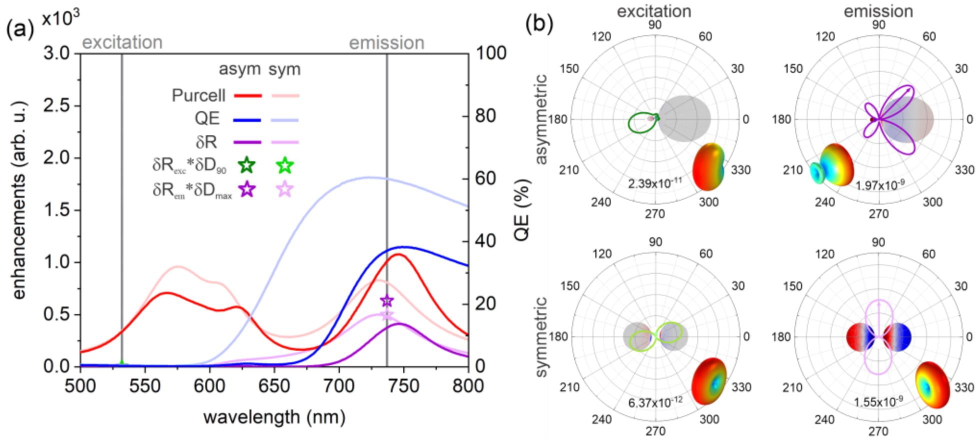

Figure S2. Optical response of optimized SiV silica-Au core-shell dimer configurations. (a) Enhancement and antenna efficiency spectra. (b) charge distribution on nanoantennae and polar angle (φ) distribution of power density radiated into far-field, and the 3D radiation pattern with rotational symmetry (the lobe direction used for directivity calculation is marked with arrow and the maximal power density radiated into this direction is indicated at the bottom of the plot).

The optimization is performed also with P_x factor as the objective function to ensure better comparability with our previous results (Figure S3) [S4, S6, S7]. In this case conditional optimization was also made, i.e. a gradually increasing minimum values of quantum efficiency were set that have to be met at the emission wavelength. The results show that the P_x factor gradually decreases as the criterion is increased, similarly to our previous optimizations [S4, S6, S7]. Important difference between silica-gold and silica-silver core-shell dimers is that with silver a significantly higher P_x factor and quantum efficiency can be achieved. The symmetric and asymmetric dimer optimizations result in coupled systems exhibiting similar P_x factor (*cQE*) tendencies, accordingly they are on the same -metal specific- branch for core-shell dimers (Figure S3).

For NV color center, the P_x factor range of $7.52 \times 10^5 - 3.26 \times 10^8$ is achieved with silica-silver core-shell nanoresonator dimers, while with silica-gold core-shell nanoresonator dimers it is orders of magnitude smaller: $2.12 \times 10^1 - 3.27 \times 10^2$. There is a small overlap between the achievable quantum efficiency regions, namely with silica-silver core-shell nanoresonator dimers 38.6% – 83.9%, while with silica-gold core-shell nanoresonator dimers 11.2% – 46% is attained. In contrast, in SiV enhancement the P_x factor region of $2.33 \times 10^7 - 1.22 \times 10^8$ (with 70.1% – 92.5% *cQE*) is attainable with silica-silver core-shell nanoresonator dimers and $2.96 \times 10^3 - 2.34 \times 10^4$ (with 25.4% – 66.7% *cQE*) is reachable with silica-gold core-shell nanoresonator dimers. These results show that in the case of SiV smaller/larger P_x factor can be achieved with silica-silver/gold compared to NV color centers, while the attainable *cQE* is larger for both metals due to the smaller material losses at the larger emission wavelength.

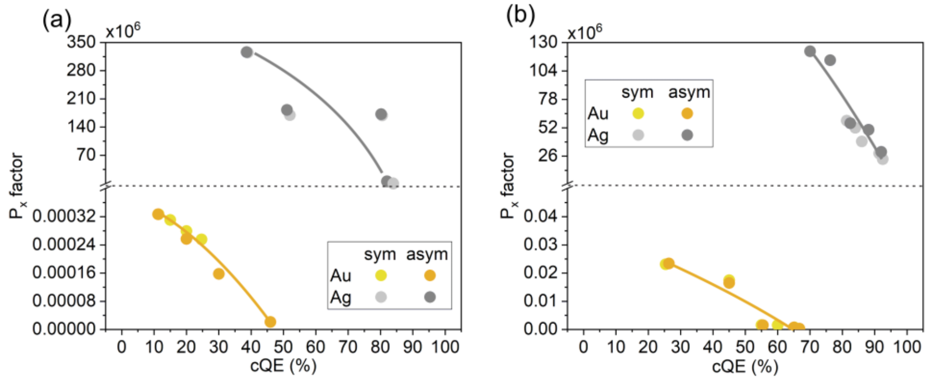

Figure S3. P_x factor of the conditionally optimized silica-silver and silica-gold core-shell dimer coupled to (a) NV and (b) SiV color centers, as a function of corrected quantum efficiency. Symmetric (sym) and asymmetric (asym) configurations are also shown.

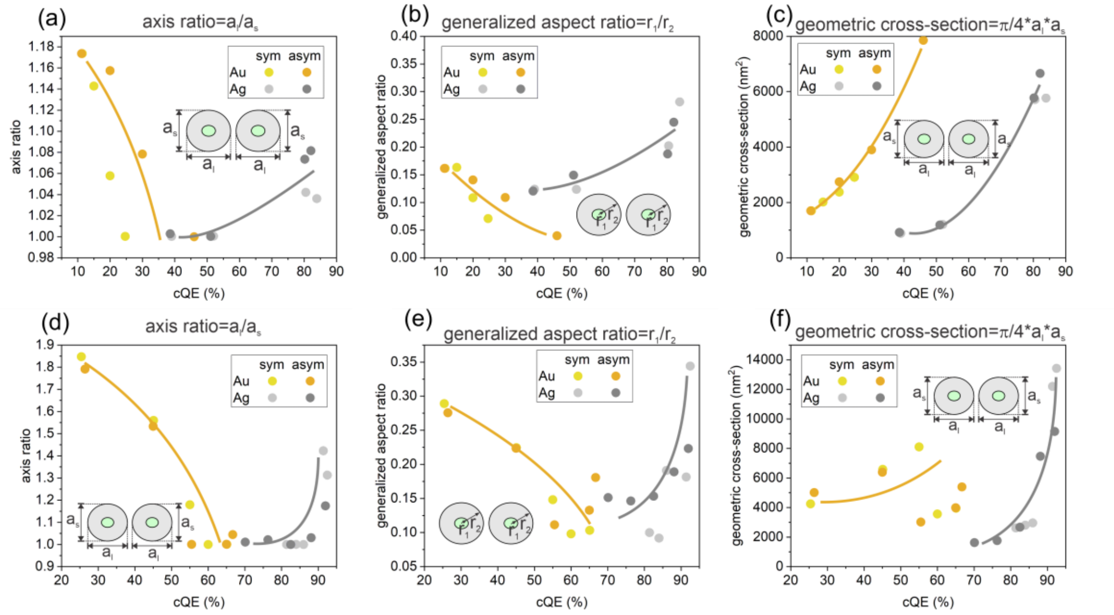

Figure S4. (a), (d) Average axis ratio (long/short axis), (b), (e) generalized aspect ratio (inner/outer radius) and (c), (f) geometric cross-section, as a function of the achieved corrected quantum efficiency (*cQE*) at the emission wavelength, in the (a)-(c) NV and (d)-(f) SiV coupled silica-gold and silica-silver core-shell dimer configurations, optimized to maximize fluorescence enhancement (P_x factor).

We have also investigated the effect of *cQE* criterion on the optimized geometry. It is found that the quantum efficiency can be increased by increasing the size of composing nanoresonators, while elongated-hollow (spherical-solid) geometries are preferred to reach high *cQE* in case of silica-silver (silica-gold) dimers (Figure S4).

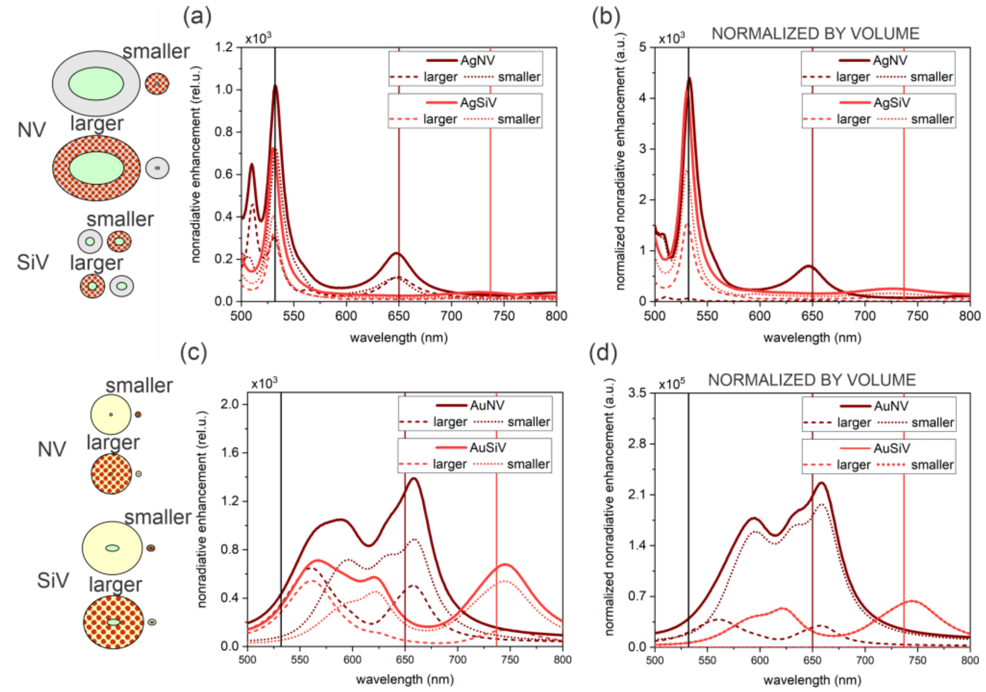

Figure S5. Nonradiative enhancement of the optimized dimers and the individual core-shell monomers composing the asymmetric dimers. (a), (b) silver, (c), (d) gold nanoshell, (a), (c) nonradiative enhancement and (b), (d) nonradiative enhancement normalized by the shell volume. The legends “smaller” and “larger” indicate the nanoshells with smaller and larger volumes, within an optimized configuration.

Figure S5 shows the nonradiative loss enhancement spectrum separately for the composing individual core-shell nanoparticles. The larger the nonradiative enhancement, the larger the resistive loss in the nanoresonator and the contribution to the antenna mode resonance. Based on all indicated spectra, the smaller nanoparticles always show larger contribution, according to the larger accumulated charge presented in the manuscript.

Examination of Figure S5 also reveals that, for silver, the maxima of the resistive heat loss spectra on the smaller and larger nanoparticles are shifted relative to each other, regardless of the color center. This shift is only a few nanometers, but the smaller (larger) nanoparticle resonates at a smaller (larger) wavelength. Exceptions are gold nanodimers at the excitation, but in those cases, only pseudomodes appear around 532 nm, as shown in Figure S1 and Figure S2. Real plasmonic modes cannot be tuned to the excitation, caused by the overlap with the electron interband transitions.

It can be concluded that by allowing smaller nanoresonator size and gap during the optimization, the resonance wavelength of plasmonic dimers can be further decreased, which would promote more accurate tuning of symmetric nanoparticles as well.

**Ag-NV**	excitation						emission						fluorescence		
	**Δλ**	**Purcell**	**δR**	**QE**	**δD_{90}**	**$\delta R \cdot \delta D_{90}$**	**Δλ**	**Purcell**	**δR**	**cQE**	**δD_{max}**	**$\delta R \cdot \delta D_{max}$**	**P_x factor**	**D_x factor**	**$D_x \cdot \delta D_{max}$**
**asym**	1	1472	474	32%	1.04	495	1	893	680	76%	2.41	1637	3.22×10^5	3.35×10^5	8.08×10^5
**sym**	2	1525	678	44%	1.36	924	79	350	310	89%	1.11	343	2.10×10^5	2.86×10^5	3.17×10^5

Table S1. Optical response of NV color center coupled, silica-silver ellipsoidal core-shell dimers, optimized to maximize D_x factor. *Δλ*: *Purcell* peak detuning (from 532 nm (excitation) and 650 nm (emission) wavelength), *Purcell* factor, *δR*: radiative rate enhancement. (*cQE*) *QE*: (corrected) quantum efficiency of the coupled system, δD_{90}: directivity enhancement in the direction of φ=90° at excitation, δD_{max}: enhancement of maximal directivity at emission, Fluorescence qualification: total fluorescence enhancement: P_x *factor* = $\delta R_{exc}\times\delta R_{em}$, projected fluorescence enhancement: D_x *factor* = $\delta R_{exc}\times\delta D_{exc}\times\delta R_{em}$ and directional fluorescence enhancements: $D_x\times\delta D_{max}$.

**Ag-SiV**	excitation						emission						fluorescence		
	**Δλ**	**Purcell**	**δR**	**QE**	**δD_{90}**	**$\delta R \cdot \delta D_{90}$**	**Δλ**	**Purcell**	**δR**	**cQE**	**δD_{max}**	**$\delta R \cdot \delta D_{max}$**	**P_x factor**	**D_x factor**	**$D_x \cdot \delta D_{max}$**
**asym**	0	940	287	31%	2.90	834	1	503	479	95%	1.04	499	1.38×10^5	3.99×10^5	4.17×10^5
**sym**	0	925	297	32%	2.88	855	1	483	461	95%	1.05	484	1.37×10^5	3.94×10^5	4.14×10^5

Table S2. Optical response of SiV color center coupled silica-silver ellipsoidal core-shell dimers optimized to maximize D_x factor. The notation is the same as in Table S1.

**Au-NV**	excitation						emission						fluorescence		
	**Δλ**	**Purcell**	**δR**	**QE**	**δD_{90}**	**$\delta R \cdot \delta D_{90}$**	**Δλ**	**Purcell**	**δR**	**cQE**	**δD_{max}**	**$\delta R \cdot \delta D_{max}$**	**P_x factor**	**D_x factor**	**$D_x \cdot \delta D_{max}$**
**asym**	57	433	0.34	0.1%	0.86	0.29	10	1609	343	21%	1.67	574	117	100	167
**sym**	66	326	0.36	0.1%	0.98	0.35	30	904	217	24%	1.05	227	78	77	80

Table S3. Optical response of NV color center coupled silica-gold ellipsoidal core-shell dimers optimized to maximize D_x factor. The notation is the same as in Table S1.

**Au-SiV**	excitation						emission						fluorescence		
	**Δλ**	**Purcell**	**δR**	**QE**	**δD_{90}**	**$\delta R \cdot \delta D_{90}$**	**Δλ**	**Purcell**	**δR**	**cQE**	**δD_{max}**	**$\delta R \cdot \delta D_{max}$**	**P_x factor**	**D_x factor**	**$D_x \cdot \delta D_{max}$**
**asym**	35	336	1.22	0.4%	0.66	0.81	9	1008	374	37%	1.69	633	456	301	509
**sym**	43	345	2.01	0.6%	0.18	0.35	7	808	485	60%	1.03	499	975	175	181

Table S4. Optical response of SiV color center coupled silica-gold ellipsoidal core-shell dimers optimized to maximize D_x factor. The notation is the same as in Table S1.

3. Benchmark of the numerical method

In order to validate the method, the results of numerical calculations were compared with analytical formulas for simpler systems, such as fluorophores located near a metal nanosphere. In this case, the non-radiative enhancement can be approximated with the formula [S8]:

$$\frac{\gamma_{nonrad}}{\gamma_{rad,0}} = \frac{3}{8k^3d^3}\mathcal{J}m\left[\frac{\varepsilon_m(\omega)-1}{\varepsilon_m(\omega)+1}\right]$$

where the transitional dipole moment of the fluorophore is perpendicular to the metal surface, k is the wavenumber, d is the distance of metal and the emitter, $\varepsilon_m(\omega)$ is the permittivity of the metal, $\gamma_{\mathrm{rad},0}$ is the emitter decay rate in free space and γ_{nonrad} is the non-radiative decay rate near the nanoparticle. The radiative rate enhancement (γ_{rad}) can be calculated using Green-functions in quasi-static approximation:

$$\frac{\gamma_{rad}}{\gamma_{rad,0}} = \left|1+2\frac{R^3}{(R+d)^3}\cdot\frac{\varepsilon_m(\omega)-1}{\varepsilon_m(\omega)+2}\right|^2$$

where R is the sphere's radius. The values obtained by the numerical method are good approximations of the analytical formulas and also reproduce the most important characteristics. As d decreases, the total and non-radiative enhancement goes to infinity, while the quantum efficiency goes to zero, as a consequence of the *QE* decrease the radiative decay is quenched (Figure S6). In the radial direction, the molecule radiates with higher efficiency near the spherical nanoparticle and the highest enhancement is obtained via the dipolar mode.

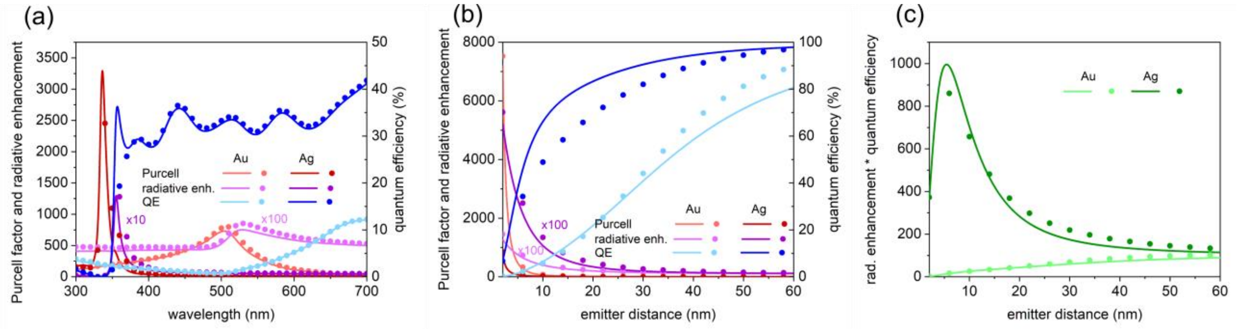

Figure S6. Optical response of an emitter near to a gold or silver nanoparticle of R=20 nm radius. (a) *Purcell* factor, quantum efficiency and radiative enhancement spectra at d=5 nm, (b) distance dependence of the Purcell factor, quantum efficiency and radiative enhancement and (c) product of radiative rate enhancement and quantum efficiency as a function of emitter distance for $\lambda_{res,Au}$=530 nm and $\lambda_{res,Ag}$=380 nm. Solid line: analytical solution, symbols: numerical solution.

It is possible to grow a diamond layer on spherical silver nanoparticles and to create a color center nanoparticle assembly [S9]. This experiment well approximates the geometry of the coupled system we have studied and the excited state lifetime of SiV color centers was 7-times shorter in the presence of nanoparticles. To benchmark our numerical method, a specific experimental geometry was reproduced. Namely, an elongated silver nanoparticle coated with diamond layer embedding a SiV color center was inspected. In order to consider the geometrical uncertainty in chemical growth, the lifetimes of several configurations were determined, allowing a standard deviation of +/-10% from the nominal geometrical values (minor axis = 300 nm, major axis = 400 nm, diamond layer thickness = 100 nm, with free variation of the color center position). In the Monte Carlo simulations, the *Purcell* factor (total decay rate change) varied between 0.06 and 320 (Figure S6). Although, the abundance of configurations with a lower Purcell factor was higher, the average decay time was found to be 0.254 ns, the corresponding calculated Purcell factor of 4.2 approximates well the experimentally determined 7-fold lifetime decrease (Figure S7).

The counterintuitive result, namely the theoretical value is smaller than the experimentally determined one, is most probably due to the fact that in reality the silver particle surface is rough. The surface roughness is capable of resulting in higher local field strength and Purcell factor due to the formation of hotspots, compared to the perfect spheroid geometry considered in theory.

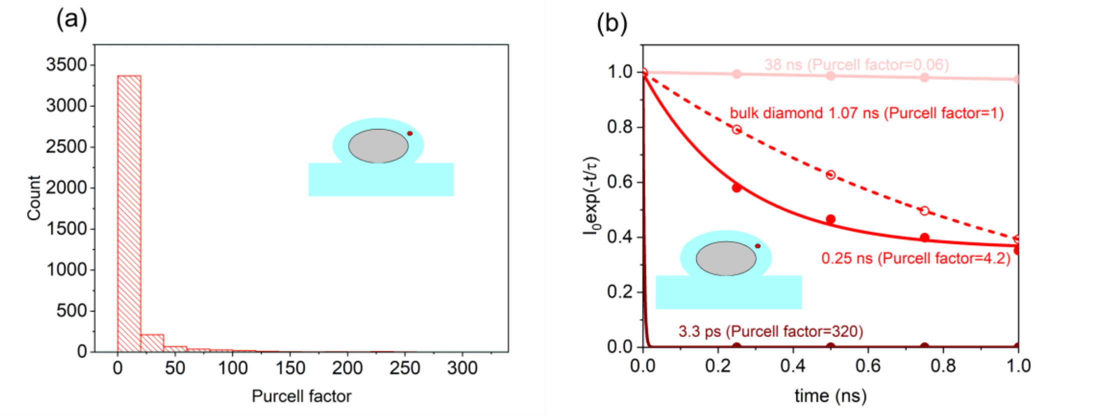

Figure S7. Number of configurations in specific *Purcell* factor intervals and the excited state lifetime of bulk diamond and assemblies with shortest, fastest and average lifetime.

References

[S1] Purcell, E. M. Spontaneous emission probabilities at radio frequencies. *Phys. Rev*. **1946**, *69*, 681.

[S2] Radko, I. P.; Boll, M.; Israelsen, N. M.; Raatz, N.; Meijer, J.; Jelezko, F.; Andersen, U. L.; Huck, A. Determining the internal quantum efficiency of shallow-implanted nitrogen-vacancy defects in bulk diamond. *Opt. Express* **2016**, *24*, 27715–27725.

[S3] Neu, E.; Agio, M.; Becher, C. Photophysics of single silicon vacancy centers in diamond: implications for single photon emission. *Opt. Express* **2012**, *20*, 19956–19971.

[S4] Szenes, A.; Bánhelyi, B.; Szabó, L. Zs.; Szabó, G.; Csendes, T.; Csete, M. Enhancing Diamond Color Center Fluorescence via Optimized Plasmonic Nanorod Configuration. *Plasmonics* **2017**, *12*, 1263–1280.

[S5] Novotny, L.; Hecht, B. Principles of Nano-Optics, 2nd ed.; Cambridge University Press: New York, 2012; pp 425–426.

[S6] Szenes, A.; Bánhelyi, B.; Szabó, L. Zs.; Szabó, G.; Csendes, T.; Csete, M. Improved emission of SiV diamond color centers embedded into concave plasmonic core-shell nanoresonators. Sci. Rep. 2017, 7, 13845.

[S7] Szenes, A.; Bánhelyi, B.; Csendes, T.; Szabó, G.; Csete, M. Enhancing Diamond Fluorescence via Optimized Nanorod Dimer Configurations. Plasmonics 2018, 13, 1977–1985.

[S8] Anger, P.; Bharadwaj, P.; Novotny, L. Enhancement and Quenching of Single-Molecule Fluorescence. *Phys. Rev. Lett.* **2006**, *96*, 113002.

[S9] Li, S.; Francaviglia, L.; Kohler, D. D.; Jones, Z. R.; Zhao, E. T.; Ogletree, D. F.; Weber-Bargioni, A.; Melosh, N. A.; Hamers R. J. Ag–Diamond Core–Shell Nanostructures Incorporated with Silicon-Vacancy Centers. ACS Materials Au 2022, 2, 85–93.
